# Acute endocrine responses to snouted cobra (*Naja annulifera*) and African puffadder (*Bitis arietans*) envenomation in dogs

**DOI:** 10.1093/jvimsj/aalag005

**Published:** 2026-02-21

**Authors:** Noeline Fourie-Viljoen, Amelia Goddard, Peter N Thompson, Sylvie Daminet, Johan P Schoeman

**Affiliations:** Department of Companion Animal Clinical Studies, Faculty of Veterinary Science, University of Pretoria, Onderstepoort, South Africa; Department of Companion Animal Clinical Studies, Faculty of Veterinary Science, University of Pretoria, Onderstepoort, South Africa; Department of Production Animal Studies, Faculty of Veterinary Science, University of Pretoria, Onderstepoort, South Africa; Vakgroep Kleine Huisdieren, Faculteit Diergeneeskunde, Universiteit Gent, Merelbeke, Belgium; Department of Companion Animal Clinical Studies, Faculty of Veterinary Science, University of Pretoria, Onderstepoort, South Africa

**Keywords:** cortisol, C-reactive protein, critical illness, snakebite, thyrotropin, thyroxine

## Abstract

**Background:**

The endocrine response to snake envenomation in dogs remains unexplored.

**Hypothesis/Objectives:**

To compare the endocrine response in dogs envenomated by snouted cobra (*Naja annulifera*) and African puffadder (*Bitis arietans*) with healthy control dogs, to evaluate the change over time of specific hormones, and to investigate the correlation of these changes with C-reactive protein (CRP).

**Animals:**

This study included 17 client-owned dogs naturally envenomed by either a snouted cobra (*N annulifera*) (*n* = 9) or a puffadder (*B arietans*) (*n* = 8). Two control groups (*n* = 10 and *n* = 12) consisted of client-owned dogs in good health.

**Methods:**

In this prospective longitudinal observational study, serum samples were collected at admission, and at 12, 24, and 36 h after envenomation. At each time point, the serum total thyroxine (TT4), thyrotropin, total cortisol, and CRP concentrations were measured.

**Results:**

The median serum TT4 concentrations of all the cases were significantly lower than those of controls at all time points (*P* < .05). The cases had a median (Q1-Q3) serum TT4 of 20.8 nmol/L (15.2-25) at admission, and 7.71 (6.4-19.7), 11.50 (6.4-18.5), and 12.3 (6.4-16.6) nmol/L at 12, 24, and 36 h after envenomation, respectively. The puffadder and neurological cobra subgroup TT4 remained significantly suppressed until 36 h after envenomation and had nadirs of 9.15 (6.4-14.65) and 6.4 (6.1-7.45) nmol/L, respectively. The non-neurological cobra subgroup had a nadir of 14.6 (9.4-21.45) nmol/L and recovered within 24 h. Serum TT4 concentration was negatively correlated with CRP (*P* < .05, ρ = −0.326). The total serum cortisol concentration in the neurological cobra subgroup at admission was 483.0 (153-549) nmol/L.

**Conclusions and clinical importance:**

Puffadder and snouted cobra envenomation are associated with profound suppression of serum TT4 concentrations, correlated with systemic inflammatory activation as measured by serum CRP concentrations.

## Introduction

Diverse causes of critical illness consistently activate several physiological processes collectively known as the “stress response” in the acute phase.^1^ The hypothalamic–pituitary–adrenal (HPA) and hypothalamic–pituitary–thyroidal (HPT) axis oscillations are central in this pursuit of homeostasis in acute critical illness.^2^ A low serum total thyroxine (TT4) concentration is characteristic in critical illness as part of the adaptive attenuation of metabolic activity in tissues, known as non-thyroidal illness syndrome (NTIS).^3^ The definition of NTIS is widely accepted as a decrease in free serum thyroid hormones in the absence of a corresponding increase in thyrotropin (TSH).^4^^,^^5^ In dogs, the suppression of TT4 concentration occurs in a plethora of disease states.^6–19^ The magnitude of this decrease in TT4 concentration is associated with different measures of severity.^6^^,^^8–10^^,^^13^^,^^19–22^ In addition, the severity or persistence of this decrease has been associated with death in parvoviral diarrhea, pancreatitis, babesiosis, and diverse non-thyroidal illness.^7^^,^^10^^,^^15^^,^^20^^,^^23^

Hypercortisolemia is typical in critically ill dogs and is associated with severity or outcome in a variety of illness states, such as sepsis, babesiosis, parvoviral enteritis, and diverse hospitalized populations.^19^^,^^20^^,^^23–27^ In human medicine, critical illness-related corticosteroid insufficiency refers to impairment of the HPA axis in certain cases of critical illness.^28^

The pathophysiology of snake envenomation is type- and degree of envenomation dependent. Snouted cobra (*Naja annulifera*) as elapid envenomation has mostly neurotoxic effects, while viperids such as puffadders (*Bitis arietans*) are associated with local cytotoxic effects and systemic vascular toxicosis,^29^ including coagulopathic effects.^30^ Inflammatory activation has been demonstrated following Puffadder^31^ and cobra envenomations in dogs.^32^

The endocrine response to snake envenomation in dogs is virtually unknown. The endocrine changes of the acute phase response are to be expected,^33^ since significant murine acute phase response activation has been established in African snake envenomation.^34^ One study found a significant increase in serum cortisol concentration in rabbits after Egyptian cobra envenomation.^35^ A subset of snake envenomed human patients also show adrenal, anterior-, and posterior pituitary dysfunction.^29^

The objectives of this study were 3-fold: first, to compare the endocrine stress response in dogs envenomed by snouted cobra and African puffadder with healthy control dogs at admission in the acute phase; second, to evaluate the change over the first 36 h of specific hormones after envenomation; and third, to investigate the correlation of these changes with the host systemic inflammatory response as indicated by C-reactive protein (CRP).

We hypothesized that (1) puffadder and snouted cobra envenomation in dogs causes a decrease in serum TT4 concentration over time in comparison to healthy controls, (2) puffadder and snouted cobra envenomation in dogs causes a rise in serum total cortisol concentration over time in comparison to healthy controls, and (3) changes in serum cortisol and TT4 concentrations in canine puffadder and snouted cobra envenomation in dogs are related to venom type.

## Materials and methods

### Study design

This prospective longitudinal observational study evaluated the data of dogs naturally envenomed by snouted cobra and African puffadder that presented to an academic hospital between November 2010 and April 2011. Previous publications on the same cohort of puffadder and snouted cobra envenomed dogs focused on the inflammatory and hemostasis variables.^32^^,^^36–38^ The endocrine assays were performed in batches after the inclusion period in February 2012 and are the first publication to utilize the endocrine data collected as part of this dataset.

### Animals

Dogs in the envenomation group were included if there was a positive identification of the snake as either a snouted cobra (*N annulifera*) or puffadder (*B arietans*). A positive identification of the snake could be obtained via photo or dead specimen, given the owner had witnessed the envenomation directly. Furthermore, the dog had to be presented within 6 h after envenomation. Additional criteria included a minimum age of 6 months and a minimum weight of 5 kg. Exclusion criteria precluded any dogs not clinically healthy, according to the owner, prior to envenomation. Any concurrent disease states or inflammatory conditions as could be established in the history or with a full physical examination, serum biochemistry, complete blood count, and blood smear resulted in exclusion. Any treatment with steroidal or nonsteroidal anti-inflammatory drugs in the month preceding envenomation also served as grounds for exclusion.

Two control groups were used. The CRP control group comprised of 10 dogs, and the endocrine control group of 12 client-owned dogs in good health, that presented for routine procedures such as blood donation or neutering, from the same background population at the same hospital. Health status was determined by owner history, physical exam, complete blood count, and serum biochemistry. The control comparisons were added because reference intervals were not recorded at the time analysis was performed, and no single historic control group with both endocrine and CRP values were available. Two separate historic control groups were thus used for statistical analysis.

Informed owner consent was obtained for participation in the study. Ethical approval was obtained from the Research Ethics Committee, Faculty of Veterinary Science, University of Pretoria (REC089-23). The original study on this cohort also obtained animal ethics approval from the Animal Ethics Committee, University of Pretoria (V058-10).

### Clinicopathological analysis

A physical examination was performed on presentation and blood samples were collected by jugular venipuncture into 3 mL ethylenediaminetetraacetic acid (EDTA) and serum vacutainer tubes using vacuum assistance. Serum samples were allowed to clot and then centrifuged within 45 min of collection at 2100*g* for 8 min. The serum samples were stored at −80°C.

The serum blood samples were collected at admission, and then at 12, 24, and 36 h after envenomation to investigate longitudinal changes. The 12-, 24-, and 36-h time points were calculated from envenomation using the time of envenomation provided by the owner during history taking.

Serum concentrations of TT4, TSH, and cortisol were measured using a previously validated^39^ solid-phase competitive chemiluminescent immunoassay, and have minimum limits of detection specific to each assay as described in Table 1. Serum concentrations below the minimum limit of detection were reported as the limit itself. Complete cell counts were performed within 30 min of admission on EDTA samples using a hematology analyzer (ADVIA 2120 Siemens). Serum CRP concentration was measured using a previously validated^40^ automated turbidimetric immunoassay that had been calibrated to ensure species-specific measurement with the heterologous assay.

**Table 1:** Calibration ranges, minimum limits of detection, and assay type performed to determine serum concentrations of TT4, TSH, total cortisol, and CRP.

	**Assay type**	**Minimum limit of detection**	**Calibration range**
**TT4**	(Canine Total T4, Siemens Medical Solutions Diagnostics, Los Angeles, USA)	6.4 nmol/L	6.4-193 nmol/L
**TSH**	(Immulite Canine TSH, Diagnostic Products Corporation, Los Angeles, USA)	0.01 ng/dL	Up to 12 ng/dL
**Total cortisol**	(Immulite 1000 Cortisol, Siemens Medical Solutions Diagnostics, Los Angeles, USA)	28 nmol/L	28-1380 nmol/L
**CRP**	Cobas Integra 400 Plus Analyzer (Roche, Basel, Switzerland)	5.1 mg/L	5.1-163.3 mg/L

Abbreviations: CRP, C-reactive protein; TSH, thyrotropin; TT4, total thyroxine.

### Treatment protocol

The clinical treatment took place in accordance with a protocol aligned with a review article by Leisewitz et al.,^31^ as far as owner finances and consent allowed. The spectrum of treatment included polyvalent antivenom, intravenous fluids (including crystalloids and colloids), blood products, and supportive treatment as necessary.

### Statistical analysis

Data is presented as median concentrations, with the IQR as indicator of data dispersion. In text where interpretive statistics are described, the mean difference and its 95% CI is given.

Statistical analysis was performed using 2 different commercial software packages, SPSS (SPSS 28.0, 2023, SPSS Inc) and Stata (StataCorp. 2023. Stata Statistical Software: Release 18. College Station, TX: StataCorp LLC). The assumption of normality on the data was tested using the Shapiro–Wilk test.

Patients were grouped on 2 levels. First, the snake species served as grounds for comparison between puffadder-envenomed and cobra-envenomed dogs, defined as the cobra (*n* = 9) and puffadder subgroups (*n* = 8). The cobra group was subdivided after initial data analysis into a group that presented with signs of neurological disease (*n* = 5) and a group without signs of neurological disease (*n* = 4). Longitudinal comparisons between dependent samples from the same group at different time points were compared using linear mixed modeling and subsequent pairwise comparisons.^41^ Once an acceptable model with the lowest Akaike information criterion was found, assumptions were checked using standard residual testing, and pairwise comparisons within this model were performed. Each time point was then compared to admission, and to every time point thereafter. Thereafter, pairwise comparisons between groups (ie, puffadder, cobra, neurological, and non-neurological groups, as well as cases and controls) within each time point were performed to evaluate time-group interactions. For all comparisons, *P* < .05 was considered significant. In addition to the dichotomous statistical significance estimation, the mean difference and 95% CI of the mean difference was reported. In addition, the precision of the data can be appreciated in the width of each 95% CI.

The correlations between CRP and TT4, CRP and cortisol, and CRP and TSH were evaluated by calculating the Spearman rank correlation coefficient, because of the nonparametric nature of the data. This correlation represents the strength of the relationship between 2 variables over all 4 time points. A Spearman’s rho value of (±)0.1-(±)0.3 was regarded a weak correlation, (±)0.4-(±)0.6 was regarded a moderate correlation, and (±)0.7-(±)0.9 was regarded a strong correlation. A *P*-value of < .05 was regarded as significant throughout.

Control dogs were established to be of similar age, sex, and breed of the study sample using ANOVA, and the control samples were all taken at a single time point.

## Results

The records of 23 naturally envenomed dogs were evaluated, and 6 dogs were excluded for incomplete data (*n* = 3), for presenting later than 6 h after envenomation (*n* = 1), and for lacking a positive identification of the snake as either snouted cobra or puffadder (*n* = 2). The weight, sex, and age of the study cohort are described in Table 2. No significant differences in age, weight, or sex were found between different groups.

**Table 2:** Descriptive statistics of the signalment of the case and control dogs. Q1 denotes the lower quartile (25th percentile), Q3 the upper quartile (75th percentile), and IQR the interquartile range. F denotes female dogs and M denotes male dogs.

	**Age (months)**	**Weight (kg)**	**Sex (*n*)**
	**Median (Q1-Q3)**	**Median (Q1-Q3)**	
**Combined cases (*n* = 17)**	43.5 (24-54.75)	11.9 (7.25-30)	F = 9; M = 8
**Puffadder group (*n* = 8)**	45 (35.5-60)	10.2 (7.55-30)	F = 3; M = 5
**Snouted cobra group (*n* = 9)**	42 (24-48)	17.8 (6.6-30.2)	F = 6; M = 3
**Neurological snouted cobra group (*n* = 5)**	48 (48-58.8)	11.2 (6-17.8)	F = 3; M = 2
**Non-neurological snouted cobra group (*n* = 4)**	24 (22.8-28.8)	31.6 (30.9-39.9)	F = 3; M = 1
**Endocrine control group (*n* = 12)**	37.50 (22.5-79.5)	27 (19-30.5)	F = 8; M = 4
**CRP control group (*n* = 10)**	51.5 (38.5-58.5)	17.6 (14.5-19.75)	F = 6; M = 4

Abbreviation: CRP, C-reactive protein.

Regarding outcome, 3 dogs did not survive to discharge. Three dogs in the neurological cobra subgroup required ventilation, of which 1 was euthanized at 72 h after admission, due to a lack of improvement despite ongoing ventilation. The 2 puffadder-envenomed mortalities were spontaneous deaths, of which one died just before 36 h after envenomation, and the other before 24 h after envenomation.

### Total thyroxine

**Figure 1:**
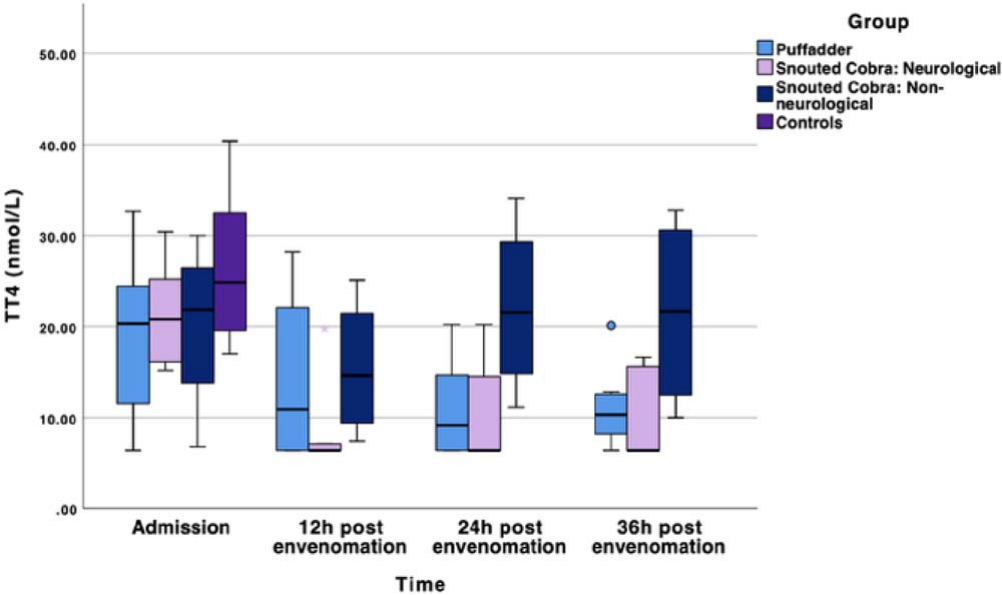
Boxplot of the median serum TT4 concentrations in nmol/L of the 3 respective snake envenomed subgroups, as well as controls. The line in the middle of the box represents the median, with the box itself representing the IQR. The whiskers represent the range by extending to the upper and lower fence values. The dots represent outliers that fall outside 1.5 times the IQR. Abbreviation: TT4 = total thyroxine.

Serum TT4 concentrations of the snake-envenomed dogs were significantly lower than that of control dogs at all time points, although the ranges of case and control serum TT4 concentrations did overlap (Figure 1; Table 3). The mean difference between controls and cases at each time point was 7.02 nmol/L (95% CI, 0.91-13.13) at admission, 14.03 nmol/L (95%CI, 8.06-20.0) at 12 h after envenomation, 13.67 nmol/L (95%CI, 7.58-19.76) at 24 h after envenomation, and 13.07 nmol/L (95%CI, 6.98-19.15) at 36 h after envenomation. For the neurological cobra subgroup, every time point from 12 h after envenomation was significantly lower than admission serum TT4 concentrations (*P* < .05). The mean difference between the admission and 12-, 24-, and 36-h serum TT4 concentration, respectively, was 12.34 nmol/L (95%CI, 7.10-17.58), 10.76 nmol/L (95%CI, 3.95-17.57), and 11.26 nmol/L (95%CI, 3.53-18.99).

**Table 3:** Descriptive statistics of serum TT4 concentrations over time. Median, lower quartile (Q1), and upper quartile (Q3) values are given in nmol/L. One asterisk (^*^) indicates a significant difference in comparison to control dogs at a significance level of *P* < .05. Two asterisks (^**^) indicate a significant difference in comparison to control dogs at a significance level of *P* < .01. “Cases” represent the combined puffadder and snouted cobra envenomation groups.

	**Time since envenomation**			
	**Admission**	**12 h postenvenomation**	**24 h postenvenomation**	**36 h postenvenomation**
	**TT4** **(nmol/L)**	**TT4** **(nmol/L)**	**TT4** **(nmol/L)**	**TT4** **(nmol/L)**
	**Median (Q1-Q3)**	**Median (Q1-Q3)**	**Median (Q1-Q3)**	**Median (Q1-Q3)**
**Combined cases (*n* = 17)**	20.80^*^ (15.2-25) (*n* = 17)	7.71^**^ (6.4-19.7) (*n* = 17)	11.50^**^ (6.4-18.5) (*n* = 16)	12.30^**^ (6.4-16.6) (*n* = 15)
**Endocrine controls (*n* = 12)**	24.85 (19.55-32.5) (*n* = 12)	–	–	–
**Puffadder (*n* = 8)**	20.30^*^ (11.55-24.4) (*n* = 8)	10.90^**^ (6.4-22.1) (*n* = 8)	9.15^**^ (6.4-14.65) (*n* = 7)	10.30^**^ (6.4-12.8) (*n* = 6)
**Snouted cobra (*n* = 9)**	20.80 (15.2-25.5) (*n* = 9)	7.71^**^ (6.4-17.8) (*n* = 9)	12.80^**^ (6.4-20.2) (*n* = 9)	15.25^**^ (6.4-22.7) (*n* = 9)
**Neurological snouted cobra (*n* = 5)**	20.8 (16.1-25.2) (*n* = 5)	6.4^**^ (6.1-7.4) (*n* = 5)	6.40^**^ (6.4-14.5) (*n* = 5)	6.40^**^ (6.4-15.6) (*n* = 5)
**Non-neurological snouted cobra (*n* = 4)**	21.85 (13.8-25.45) (*n* = 4)	14.6^*^ (9.4-21.45) (*n* = 4)	21.55 (14.8-19.35) (*n* = 4)	21.65 (12.45-30.6) (*n* = 4)

Abbreviation: TT4, total thyroxine.

At the 12-h time point, the serum TT4 concentrations of both the combined cases and the non-neurological cobra subgroup reached its nadir.

At 24 h after envenomation, the overall median TT4 concentration of the study cohort had started to increase but was still significantly lower than admission (mean difference −6.58 nmol/L [95%CI, −9.73 to −3.43]; *P* < .05) and the controls (mean difference −13.67 nmol/L [95%CI, −19.76 to −7.58]; *P* < .001). The non-neurological cobra subgroup’s median TT4 serum concentration had started to increase in comparison to the 12-h time point (mean difference 6.65 nmol/L [95%CI, 0.79-12.51]; *P* < .05) and was no longer significantly lower than the controls. The puffadder subgroup’s nadir at 24 h after envenomation was significantly lower than admission (mean difference −8.08 nmol/L [95%CI, −13.46 to −2.69]; *P* < .05), and the controls (mean difference −15.88 nmol/L [95%CI, −23.08 to −8.68]; *P* < .001). At this point, the non-neurological cobra subgroup serum TT4 concentration was significantly higher than that of both the puffadder (mean difference 11.2 nmol/L [95%CI, 1.64-20.76]; *P* < .05) and neurological cobra (mean difference 11.30 nmol/L [95%CI, 0.82-21.77]; *P* < .05) groups.

At 36 h after envenomation, the combined cases (mean difference −5.97 nmol/L [95%CI, −9.49 to −2.46]), neurological cobra (mean difference −11.26 nmol/L [95%CI, −18.99 to −3.53]) and puffadder (mean difference −8.22 nmol/L [95%CI, −14.50 to −1.94]) subgroup serum TT4 concentrations were still significantly lower than admission (*P* < .05). Both the neurological cobra (mean difference −14.41 nmol/L [95%CI, −22.30 to −6.52]) and puffadder (mean difference −16.10 nmol/L [95%CI, −23.39 to −8.81]) subgroups were still significantly lower than controls at 36 h after envenomation (*P* < .001). In contrast, for the non-neurological cobra subgroup the serum TT4 concentration had increased significantly compared to the 12-h time point (*P* < .05) and was still significantly higher than that of the puffadder (mean difference 10.79 nmol/L [95%CI, 1.13-20.45]) and neurological cobra (mean difference 11.25 nmol/L [95%CI, 0.77-21.72]) subgroups (*P* < .05). No difference was detected between non-neurological cobras and controls at this time point.

### Thyroid-stimulating hormone

**Figure 2:**
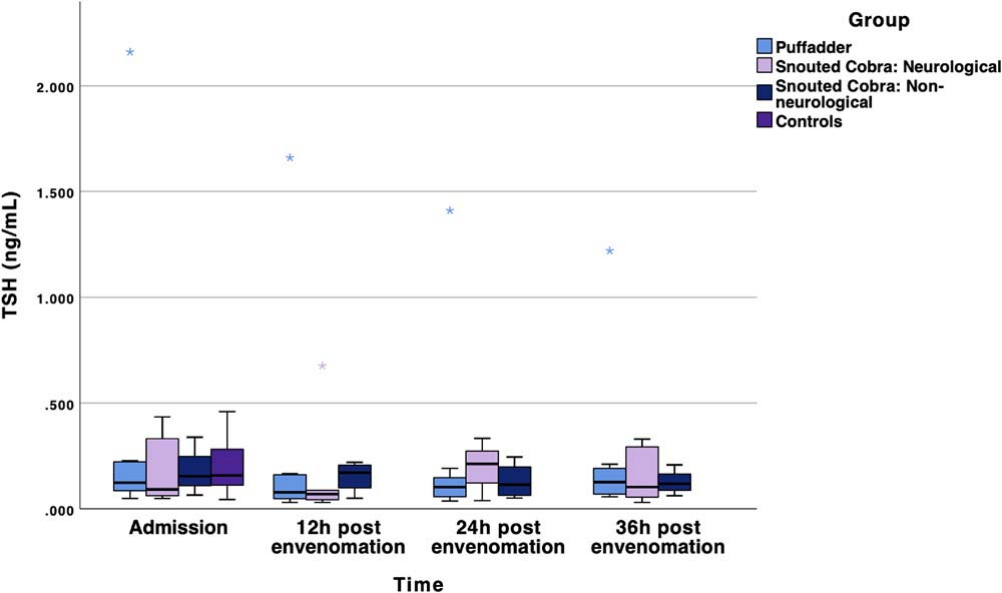
A clustered boxplot of serum TSH concentrations (in ng/mL) at admission, and at 12, 24, and 36 h post envenomation. The line in the middle of the box represents the median, with the box itself representing the IQR. The whiskers extend to the upper and lower fence values. The dots represent outliers that fall outside 1.5 times the IQR. Abbreviation: TSH = thyrotropin.

When comparing slightly lower serum TSH concentrations of cases to that of control dogs, the comparisons failed to reach significance (Figure 2). The mean difference in the neurological cobra group between 24 and 12 h after envenomation was 0.01 ng/mL (95%CI, −0.10 to 0.07), and in the non-neurological cobra group the mean difference between 12 h after envenomation and admission was 0.03 ng/mL (95%CI, −0.11 to 0.16; Table 4; Figure 2). In the puffadder subgroup, admission serum TSH concentrations were higher than 12 (mean difference 0.1 ng/mL [95%CI, 0.01-0.20]), 24 (mean difference 0.13 ng/mL [95%CI, 0.02-0.23]), and 36 h (mean difference 0.14 ng/mL [95%CI, 0.01-0.26]) after envenomation. Extreme outliers were present in the TSH data, which likely ties in with the low sensitivity of the assay, as discussed below.

**Table 4:** Descriptive statistics of serum TSH concentrations over time. Median, lower quartile (Q1), and upper quartile (Q3) values are given in ng/mL. One asterisk (^*^) indicates a significant difference in comparison to control dogs at a significance level of *P* < .05. Two asterisks (^**^) indicate a significant difference in comparison to control dogs at a significance level of *P* < .01. “Cases” represent the combined puffadder and snouted cobra envenomation groups.

	**Time since envenomation**			
	**Admission**	**12 h post envenomation**	**24 h post envenomation**	**36 h post envenomation**
	**TSH** **(ng/mL)**	**TSH** **(ng/mL)**	**TSH** **(ng/mL)**	**TSH** **(ng/mL)**
	**Median (Q1-Q3)**	**Median (Q1-Q3)**	**Median (Q1-Q3)**	**Median (Q1-Q3)**
**Combined cases (*n* = 17)**	0.12 (0.07-0.23) (*n* = 17)	0.08 (0.05-0.17) (*n* = 17)	0.10 (0.05-0.21) (*n* = 16)	0.11 (0.06-0.21) (*n* = 15)
**Endocrine controls (*n* = 12)**	0.16 (0.11-0.28) (*n* = 12)	–	–	–
**Puffadder (*n* = 8)**	0.124 (0.09-0.22) (*n* = 8)	0.08 (0.05-0.16) (*n* = 8)	0.10 (0.06-0.15) (*n* = 7)	0.13 (0.06-0.21) (*n* = 6)
**Snouted cobra (*n* = 9)**	0.12 (0.06-0.33) (*n* = 9)	0.08 (0.04-0.19) (*n* = 9)	0.14 (0.05-0.25) (*n* = 9)	0.11 (0.06-0.21) (*n* = 9)
**Neurological snouted cobra (*n* = 5)**	0.09 (0.06-0.33) (*n* = 5)	0.07 (0.04-0.09) (*n* = 5)	0.21 (0.12-0.27) (*n* = 5)	0.10 (0.06-0.29) (*n* = 5)
**Non-neurological snouted cobra (*n* = 4)**	0.16 (0.11-0.25) (*n* = 4)	0.171 (0.01-0.21) (*n* = 4)	0.114 (0.06-0.20) (*n* = 4)	0.12 (0.09-0.17) (*n* = 4)

Abbreviation: TSH, thyrotropin.

### Cortisol

**Figure 3:**
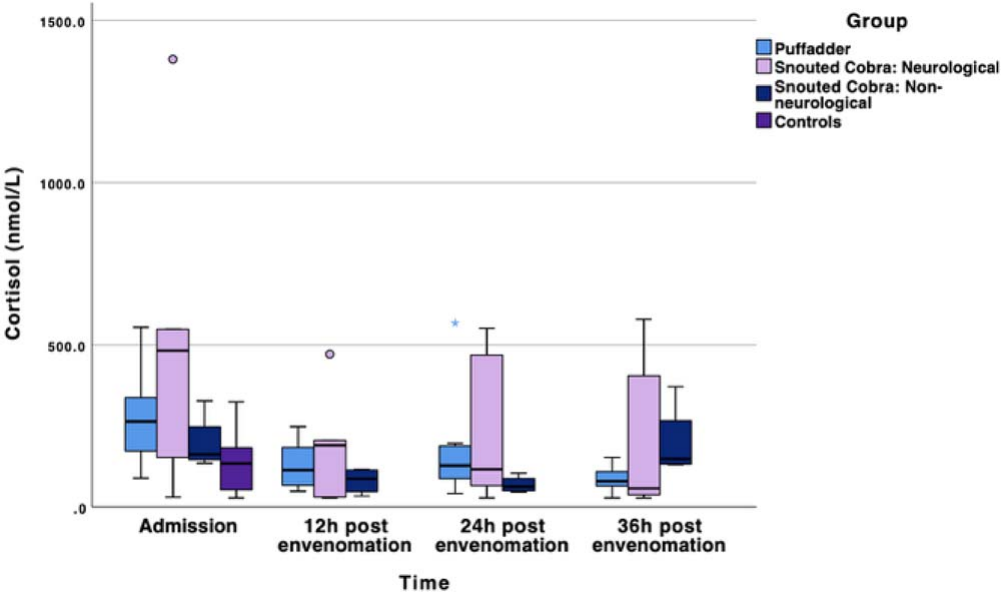
Boxplot of the median serum cortisol concentrations in nmol/L of the 3 respective snake envenomed subgroups, as well as controls. The line in the middle of the box represents the median, with the box itself representing the IQR. The whiskers represent the range by extending to the upper and lower fence values. The dots represent outliers that fall outside 1.5 times the IQR.

At admission, the median serum total cortisol concentration of the combined cases was at its highest (Table 5). The mean difference in median serum cortisol concentration of cases compared to controls at admission was 170.16 nmol/L (95%CI, −20.44 to 360.75). At 12 (mean difference −183.13 nmol/L [95%CI, −285.72 to −80.55]) (*P* < .05), 24 (mean difference −147.17 nmol/L [95%CI, −234.27 to −60.07]; *P* < .05), and 36 (mean difference −160.39 nmol/L [95%CI, −243.50 to −77.26]) h after envenomation (*P* < .01; Figure 3) the serum total cortisol concentrations of the combined cases were significantly lower than at admission. In the puffadder subgroup, the serum total cortisol concentrations at 36 h after envenomation were significantly lower than at admission (mean difference −182.67 nmol/L [95%CI, −340.47 to −24.86]; *P* < .01). The neurological cobra subgroup’s serum total cortisol concentrations were significantly lower at the 12- (mean difference −333.54 nmol/L [95%CI, −600.95 to −66.13]; *P* < .01), 24- (mean difference −272.56 nmol/L [95%CI, −500.31 to −44.81]; *P* < .05), and 36-h (mean difference −297.2 nmol/L [95%CI, −495.31 to −99.09]; *P* < .01) time points compared to admission.

**Table 5:** Descriptive statistics of serum cortisol concentrations over time. Median, lower quartile (Q1), and upper quartile (Q3) values are given in nmol/L. One asterisk (^*^) indicates a significant difference in comparison to control dogs at a significance level of *P* < .05. Two asterisks (^**^) indicate a significant difference in comparison to control dogs at a significance level of *P* < .01. “Cases” represent the combined puffadder and snouted cobra envenomation groups.

	**Time since envenomation**			
	**Admission**	**12 h postenvenomation**	**24 h postenvenomation**	**36 h postenvenomation**
	**Cortisol** **(nmol/L)**	**Cortisol** **(nmol/L)**	**Cortisol** **(nmol/L)**	**Cortisol** **(nmol/L)**
	**Median (Q1-Q3)**	**Median (Q1-Q3)**	**Median (Q1-Q3)**	**Median (Q1-Q3)**
**Combined cases (*n* = 17)**	227.50 (153-361) (*n* = 17)	108.00 (49.1-191) (*n* = 17)	111.00 (54.4-180) (*n* = 16)	85.80 (58.2-153) (*n* = 15)
**Endocrine controls (*n* = 12)**	135.00 (53.7-183) (*n* = 12)	–	–	–
**Puffadder (*n* = 8)**	264.5 (173-338) (*n* = 8)	114.5 (68-184) (*n* = 8)	128.5 (88.3-188.5) (*n* = 7)	80.3 (63.7-134) (*n* = 6)
**Snouted cobra (*n* = 9)**	163.0 (136-483) (*n* = 9)	87.6 (30.9-191) (*n* = 9)	69.8 (46.9-117) (*n* = 9)	133.0 (38.4-372) (*n* = 9)
**Neurological snouted cobra (*n* = 5)**	483.0 (153-549) (*n* = 5)	191 (30.9-206) (*n* = 5)	117.0 (66.8-469) (*n* = 5)	58.2 (38.4-406) (*n* = 5)
**Non-neurological snouted cobra (*n* = 4)**	163 (146.5-248.5) (*n* = 4)	87.6 (47.7-115.5) (*n* = 4)	63.6 (50.7-88.9) (*n* = 4)	148.5 (133-267) (*n* = 4)

The individual case with the highest serum total cortisol concentration at 36 h after envenomation (579 nmol/L), as well as the highest serum CRP concentration (197.3 mg/L), also had the lowest sustained serum TT4 concentration (below the minimum limit of detection) at 36 h after envenomation.

There was 1 hypocortisolemic dog in the study cohort. The 5-year-old Staffordshire bull terrier was envenomed by a snouted cobra and had serum total cortisol concentrations below the minimum limit of detection at every time point except at admission, where it had a concentration of 30.6 nmol/L. It presented with respiratory paralysis, was manually ventilated, failed to show overt hypoglycemia or hypotension and survived to discharge despite consistent hypocortisolemia for the duration of the study period. This dog had a Na:K ratio of 31.1:1 and urine specific gravity of 1.050 at admission and no other clinical or anamnestic indication of pre-existing hypoadrenocorticism.

### C-reactive protein

**Figure 4:**
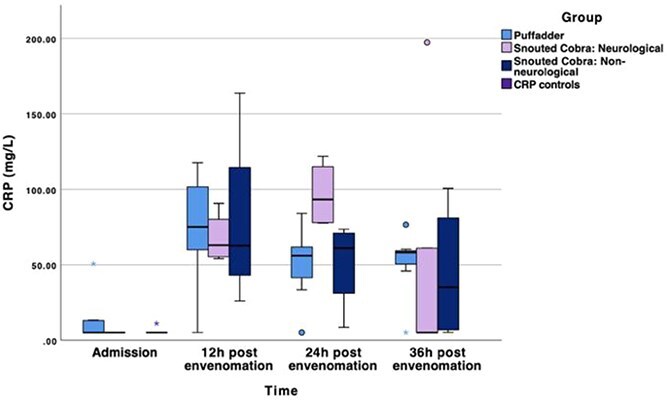
Boxplot of the median serum CRP concentrations in mg/L of the 3 respective snake envenomed subgroups, as well as controls. The line in the middle of the box represents the median, with the box itself representing the IQR. The whiskers represent the range by extending to the upper and lower fence values. The dots represent outliers that fall outside 1.5 times the IQR. Abbreviation: CRP = C-reactive protein.

The CRP of the combined cases was significantly higher than that of the control group at 12 (mean difference 76.82 mg/L [95%CI, 43.02-110.62]; *P* < .001), 24 (mean difference 56.50 mg/L [95%CI, 36.67-76.33]; *P* < .001), and 36 (mean difference 44.25 mg/L [95%CI, 12.26-76.23]; *P* < .01) h after envenomation, but not at admission (Table 6). The neurological cobra subgroup peak in CRP serum concentrations at 24 h after envenomation was significantly higher than the puffadder (mean difference 41.87 mg/L [95%CI, 10.73-73.01]; *P* < .001) and non-neurological cobra (mean difference 41.80 mg/L [95%CI, 5.45-78.14]; *P* < .01) groups at that time point (Figure 4). The puffadder and neurological cobra subgroup median serum CRP concentrations were also significantly higher than the controls at 12 (mean difference puffadder 68.8 mg/L [95%CI, 24.93-112.68], mean difference neurological cobra 97.73 mg/L [95%CI, 42.63-144.82]; *P* < .01), 24 (mean difference puffadder 45.25 mg/L [95%CI, 26.48-64.01], mean difference neurological cobra 86.94 mg/L [95%CI, 64.64-109.24]; *P* < .001), and 36 (mean difference puffadder 46.66 mg/L [95%CI, 6.83-86.50], mean difference neurological cobra 49.00 mg/L [95%CI, 3.30-94.70]; *P* < .05) h after envenomation. The non-neurological cobra subgroup was only significantly higher than the controls at 12 (mean difference 73.10 mg/L [95%CI, 18.37-127.82]; *P* < .05) and 24 (mean difference 45.32 mg/L [95%CI, 21.92-68.73]; *P* < .001) h after envenomation (Figure 4).

**Table 6:** Descriptive statistics of serum CRP concentrations over time. Median, lower quartile(Q1), and upper quartile(Q3) values are given in mg/L. One asterisk (^*^) indicates a significant difference in comparison to control dogs at a significance level of *P* < .05. Two asterisks (^**^) indicate a significant difference in comparison to control dogs at a significance level of *P* < .01. “Cases” represent the combined puffadder and snouted cobra envenomation groups.

	**Time since envenomation**			
	**Admission**	**12 h post envenomation**	**24 h post envenomation**	**36 h post envenomation**
	**CRP** **(mg/L)**	**CRP** **(mg/L)**	**CRP** **(mg/L)**	**CRP** **(mg/L)**
	**Median (Q1-Q3)**	**Median (Q1-Q3)**	**Median (Q1-Q3)**	**Median (Q1-Q3)**
**Combined cases (*n* = 17)**	5.10 (5.10-8.93) (*n* = 11)	64.68^**^ (57.86-90.18) (*n* = 16)	61.73^**^ (51.58-77.94) (*n* = 16)	56.67^*^ (5.10-61.24) (*n* = 16)
**Controls (*n* = 10)**	5.10 (5.10-5.10) (*n* = 10)	–	–	–
**Puffadder (*n* = 8)**	264.5 (173.0-338.0) (*n* = 7)	114.5^**^ (68.0-184.0) (*n* = 8)	128.5^**^ (88.3-188.5) (*n* = 8)	80.3^*^ (63.7-134.0) (*n* = 7)
**Snouted cobra (*n* = 9)**	5.10 (5.10-5.10) (*n* = 4)	87.6^**^ (30.9-191.0) (*n* = 8)	69.8^**^ (46.9-117.0) (*n* = 8)	133.0^*^ (38.4-372.0) (*n* = 9)
**Neurological snouted cobra (*n* = 5)**	5.10 (5.10-5.10) (*n* = 4)	62.98^**^ (55.32-80.07) (*n* = 4)	93.27^**^ (77.94-115.03) (*n* = 4)	5.10^*^ (5.10-60.96) (*n* = 5)
**Non-neurological snouted cobra (*n* = 4)**	– (*n* = 0)	62.68^*^ (43.09-114.51) (*n* = 4)	60.99^**^ (31.08-70.97) (*n* = 4)	35.09 (6.88-81.06) (*n* = 4)

Abbreviation: CRP, C-reactive protein.

A weak yet significant negative correlation between serum TT4 and CRP concentrations was found (ρ = −0.326; *P* < .05). The correlation between serum total cortisol and CRP concentrations was not significant.

## Discussion

This study presents a description of the longitudinal changes in serum TT4, TSH, and total cortisol concentrations in dogs envenomed by puffadder and snouted cobra envenomation over the first 36 h after envenomation. A significant suppression of serum TT4 concentrations was observed at all time points in the study cohort. In addition, the association of TT4 suppression and degree of systemic inflammation as measured by serum CRP concentration has not previously been demonstrated in dogs. The longitudinal thyroid hormone and cortisol changes seen in puffadder and snouted cobra envenomation are rapid, reflecting a similarity to endotoxaemia^11^ in contrast to most longitudinal endocrine studies that have been performed on infectious diseases.^10^^,^^19^^,^^23^^,^^24^

Total thyroxine changes induced by puffadder and snouted cobra envenomation were characterized by a significant suppression in comparison to control dogs at every time point. This demonstrates a longer lasting initial suppression in the acute phase than after repeated dosages of 1 μg/kg *Escherichia coli* O111:B4 endotoxin administered intravenously every 8 h for 84 h, where the serum TT4 concentration only showed significantly lower concentrations at 8 and 12 h after initiating endotoxin administration.^11^ The non-neurological cobra subgroup deviated from this pattern, with an early normalization of TT4 concentrations between 12 and 24 h after envenomation. In this non-neurological group as a lower severity subgroup, the early recovery from NTIS might indicate that sustained TT4 suppression is associated with severity of the underlying condition.

The onset of TT4 suppression in puffadder and snouted cobra envenomation was similar to that reported in a dog bite study with a nadir at 16 h, but the suppression was sustained for longer, only reaching the normal reference interval by 72 h.^16^ Another study on diverse mild nonthyroidal illness reported the lowest TT4 concentrations at admission, but the time since onset of disease could not be determined, in contrast to envenomation where the time of onset of disease is often known.^12^ In experimental babesiosis, the first significant decrease compared to controls was only seen at 72 h after infection.^19^ Although this infectious disease model seems to have a later nadir in TT4 concentrations, the time taken for parasitemia to develop after experimental inoculation must be factored in.

In this study, there was a weak significant negative correlation between TT4 and CRP concentrations; however, the TT4 suppression preceded the first spike in CRP, with the TT4 already demonstrating significant suppression in comparison to control dogs at admission, and the CRP only increasing significantly at 12 h after envenomation. The CRP pattern is not unexpected given the kinetics of the inflammatory response. This CRP response reported here is slower than that to surgical trauma or to experimental injection of *E coli* lipopolysaccharide, which is detected as early as 4 h.^42^ The peak at 12-24 h after envenomation is more comparable to surgery,^43^ and gastric dilatation volvulus.^44^ Another longitudinal study on parvoviral enteritis found a negative correlation between TT4 and the presence of systemic inflammatory response syndrome.^10^ The negative correlation demonstrates that the progression of NTIS is correlated to the severity of the host inflammatory response. Experimental studies indicate that inflammation is key to NTIS progression.^3^^,^^45–47^ Inflammatory markers and NTIS are associated with severity in sepsis.^48–50^ One pediatric study reported a significant negative correlation between free triiodothyronine and CRP.^51^ Puffadder envenomations cause inflammatory activation.^37^^,^^52^^,^^53^ In our study, the increase in CRP, as well as the decrease in TT4 were, however, more pronounced in the neurological cobra subgroup, considered a clinically severe subgroup. Similar patterns are seen in other studies, where sustained suppression of TT4 seems to be present in groups with more severe disease.^19^^,^^23^

In addition to the marked suppression of TT4, much is to be said for the TT4–TSH dissociation. While some experimental studies demonstrated a suppression in TSH after interleukin injection as simulation of inflammation,^5^^,^^54^^,^^55^ little clinical evidence is available. In dogs with bite wounds, serum TSH concentrations remain within the reference interval until 56 h after the injury, whereafter TSH increases.^16^ It is possible that our study lacked the sample size to detect the significance of an increase in TSH concentration, but more likely that the duration of the study was not long enough, or that peripheral mechanisms are responsible for TT4 suppression in snake envenomed dogs.

Only the neurological cobra subgroup at admission showed significant elevation in total cortisol concentration compared to the control group. The median total cortisol concentration at the highest point, admission, was 483 nmol/L in the neurological cobra subgroup, and 264.5 nmol/L in the puffadder group. Dogs with bite wounds, experimental babesiosis, and sepsis induced by *Staphylococcus pneumoniae* have peak median total cortisol concentrations of 314.6, 315, and > 400 nmol/L, respectively.^16^^,^^19^^,^^24^ Anecdotally, the patient with the highest sustained total cortisol concentration (579 nmol/L), also had the highest CRP concentration at time point 36 h (197.3 mg/L), and the lowest TT4 concentration (below the minimum limit of detection). In the experimental babesiosis study, the single death also had the highest total cortisol concentration of 610 nmol/L.^19^ Hypercortisolemia is a consistent finding during the acute phase of critical illness, that is proportional to severity,^19^^,^^23–26^^,^^56–58^ as was corroborated in the cortisol responses of snake-envenomed dogs in this study.

There were several limitations in our study. The size of the study was compensated for by investigating multiple pairwise comparisons in an attempt to balance the risk of types 1 and 2 statistical error. This makes it likely that all clinically significant changes within the investigated timeframe were reported. The screening of dogs for concurrent illnesses was limited and depended largely on owner history, since blood results are affected by the envenomation itself. Being a post-hoc analysis of prospectively collected data, most constraints of the age of the data could be mitigated. The lack of reference ranges that stems from this post-hoc analysis is also a limitation, since the reference ranges for the assays performed were no longer available by the time of data analysis. This led to the inclusion of control groups. The study was also limited by its nonexperimental nature, as owner finances and consent limited the protocol. Another limitation was that a snake bite severity score had not been incorporated into the original study design, which could have added a correlation of endocrine variables with severity of envenomation. The second-generation TSH assay used is known to lack the sensitivity required to detect fine oscillations in the HPT axis during critical illness. Missing data were also a limitation present in this study due to its clinical nature. Mortalities earlier in the study resulted in missing data at the later time points, and CRP values were missing for many individuals at the first time point. Statistical methods that are proven robust in clinical settings with missing data were used to minimize the impact of missing datapoints. The control groups were also limited, as ideally one would not only be able to compare snake envenomed animals to healthy controls but also to sick controls with an unrelated illness or injury, such as trauma. In that way, the homogeneity of the endocrine response to illness in general could be contrasted with disease-specific changes. Control groups were preferred to reference intervals, as control groups allowed for interpretive statistics to be performed. Another significant limitation is the variable time periods between envenomation and presentation. The median interval between envenomation and presentation in the puffadder and cobra group was 1.5 (1-2) and 3 (1-4.5) h, respectively. After admission, all time points were calculated from the time of observed envenomation, and not from admission. This enabled the variance to be limited to the first time point and not repeated at and between time points 12, 24, and 36 h. The exclusion of dogs presenting over 6 h after envenomation excluded some higher severity cases referred from other institutions at a later stage, which could have resulted in higher severity endocrine patterns being missed.

Puffadder (*B arietans*) and snouted cobra (*N annulifera*) envenomations cause significant endocrine disturbances in dogs. The most severe perturbations in both the HPA and the HPT axes were seen in a subgroup of snouted cobra envenomed dogs showing signs of neurological disease.

## Off-label antimicrobial declaration

The authors declare no off-label use of antimicrobials.

## Institutional animal care and use committee or other approval declaration

Approved by the Research Ethics Committee, Faculty of Veterinary Science, University of Pretoria (REC089-23). The original study on this population also obtained animal ethics approval from the Animal Ethics Committee, University of Pretoria (V058-10).

## Abbreviations

TT4: total thyroxine
TSH: thyrotropin
CRP: C-reactive protein
HPA: hypothalamic–pituitary–adrenal
HPT: hypothalamic–pituitary–thyroidal
NTIS: non-thyroidal Illness syndrome
